# 

**Published:** 1896-08-29

**Authors:** 


**Notices of Births, Deaths, and Marriages** are inserted in this column at a charge of 2s. 6d. for an announcement not exceeding four lines.

### NOTICE TO CORRESPONDENTS.

All MS., Letters, Books for Review, and other matters intended for the Editor should be addressed The Editor, The Lodge, Porchester Square, London, W.

The Editor cannot undertake to return rejected MS., even when accompanied by stamped directed envelope.

### Notice to Advertisers and Subscribers.

All Advertisements, Orders for Copies of The Hospital and Business Communications should be addressed to **THE MANAGER, (Not to The Editor),** The Hospital, 428, Strand, London, W.C.

The Cost of Subscription, post free, is as follows:—Per week, 2½d.; per quarter, 2s. 8d.; per half-year, 5s. 3d.; per annum, 10s. 6d. Foreign:—Per Annum, 15s. 2d.; payable, in all cases, in advance.

Price 5s.

**"Burdett's Hospitals and Charities, 1896."**

Seventh year of publication. Over 1,000 pp. Scarlet cloth, gilt lettered, Price 5s. A most complete guide to British, Colonial, Indian, and American Hospitals, Dispensaries, Nursing and Convalescent Institutions, and Asylums. A few complete sets of the preceding six issues of the "Annual" may still be obtained on application to the Publishers, The Scientific Press (Limited), 428, Strand, London, W.C.

### SCRAPS AND GLEANINGS.

Two isolation wards are to be added to the Hereford Infirmary.

Two new branches of the Newcastle Dispensary were opened recently.

The institution of Hospital Saturday at Newark is under consideration.

It is proposed to spend £1,200 on a scheme for heating the Royal Berkshire Hospital at Reading.

The Hospital Saturday collection in aid of the Victoria Hospital, Lewes, has realised £158 this year.

The income of the Forgue Cottage Hospital for 1895-6 amounted to £122, and the expenditure to £125.

Madame Patti has decided not to give a concert this year in aid of the funds of the Swansea Hospital.

The Balmain Cottage Hospital, New South Wales, is to be enlarged by the addition of a one-storey building.

Sir W. T. Lewis, Bart., has given £1,000 towards the construction of an accident ward for the Merthyr General Hospital.

At the Ancoats Hospital, Manchester, 1,061 in, 6,316 home, 2,205 out, and 291 dental patients were treated during the year just ended.

An anonymous gift of £500 has been received by the Deaconess' Hospital Board, Edinburgh, towards the hospital building extension fund.

It is stated that the amount raised by the Ladies' Committee of Mrs. Lucas Shadwell's scheme in aid of the Hastings, St. Leonard's and East Sussex Hospital will be over £900.

The expenditure at the Shirley Children's Hospital during the past year has been nearly double the income, viz., £230, against an income of £130. Fifty in and 360 out patients were treated.

Of the £585 received during 1895 by the Alexandra Hospital, Woodhall Spa, £282 was obtained from patients' fees. The expenditure amounted to £592. One hundred and forty-one patients were treated in 1895, compared with 129 in 1894.

The question of providing isolation hospital accommodation is attracting serious attention in the counties. Several county and district councils have been considering the advisability of providing joint accommodation for infectious diseases.

At the Heatherdene Convalescent Home, a branch of the Sunderland Infirmary, the patients cost on an average for maintenance during the year 1895-6 11s. 7d. a week. 242 patients were sent from the Infirmary to the home, against 235 in the previous year.

Twenty-four more beds (making 40 in all) will soon be added to the Monkwearmouth and Southwick Hospital. During the year just ended 212 in-patients were treated, as against 195 in the previous year, and 4,181 out-patients. The largest number of accidents treated in any one month was 501.

The amount received on behalf of the Carlisle Hospital Sunday Fund this year was £647, and of the Hospital Saturday Fund £432. Of these amounts Carlisle contributed £176 to the Sunday and £371 to the Saturday Fund, Cumberland £402 and £53 respectively, and the balance in each case was received from Westmoreland.

Owing to the Vicar of Gorleston refusing to allow a collection in aid of the Gorleston Cottage Hospital to be made in his church, the Friendly Societies of the neighbourhood have for the past two years held their annual Sunday demonstration in aid of the hospital on the beach. A service has been held and a sermon preached in an extemporised pulpit made of fish trunks covered with red and white bunting.

During the year ended June 30th, 1896, 2,417 in and 5,434 out-patients were treated at the Sunderland Infirmary, being increases when compared with the previous year of 55 and 581 respectively. The amount of annual subscriptions received (£1,714) show an increase of £135, and the receipts from workpeople (£4,950) an increase of nearly £250 over the previous year, a fact which the committee refer to with much satisfaction.

Two of the presents received by the Clayton Hospital, Wakefield, are noteworthy. One firm has for some years supplied as a gift the soap required for the use of the hospital; and another firm has this year replaced the hempen ropes for the lift with steel ones free of cost. Three thousand six hundred and forty-eight out and 697 in-patients were treated during the year ended June 30th, 1896; while 835 patients were visited in their own homes.

The Guildford Hospital Saturday Committee have generously decided not to use the letters which they receive as a result of their collections in aid of the Royal Surrey County Hospital and the Surrey Convalescent Home. This makes their collection really a gift to the hospital. It has been calculated that if the whole of the letters sent to the committee were used it would cost the hospital £300, while the collections in respect of which they are given only amount to £120.

### THE STUDENT OF MEDICINE.

### APPOINTMENTS.

D. Campbell Black, M.B.Glasg., L.R.C.S.Edin., appointed Examiner in Physiology in the University of St. Andrews. F. J. Burman, L.R.C.P.Edin., M.R.C.S.Eng., appointed Medical Officer for the Wath District of the Rotherham Union. R. J. Burns, M.R.C.S.Eng., L.R.C.P.Lond., appointed Medical Officer for the Bishopwearmouth East District of the Sunderland Union. C. W. Eames, M.R.C.S., L.R.C.P.Lond., appointed Assistant Medical Officer to the Workhouse of the Leeds Union. W. Willoughby Gardner, M.D.Lond., M.R.C.S.Eng., L.R.C.P., appointed Physician to the Salop Infirmary. F. W. Gibbon, L.R.C.P., L.R.C.S.Edin., appointed Medical Officer for the First District and the Workhouse of the St. Albans Union. J. Lee, M.B., C.M.Edin., appointed Junior House Surgeon to the District Hospital, West Bromwich. A. Leigh, M.B., Ch.B.Vict., appointed Medical Officer for the Witton District of the Blackburn Union. G. Lys, L.R.C.P.Lond., M.R.C.S.Eng., appointed Medical Officer for the Almer District of the Wimborne and Cranborne Union. J. F. Pridie, M.B., C.M.Edin., appointed Medical Officer for the Chingford District of the Epping Union. J. R. Prior, M.B.Durh., M.R.C.S., L.R.C.P., appointed Junior House Surgeon to the Seamen's (late "Dreadnought") Branch Hospital, Royal Albert Docks, E. Samuel Prior, M.B., C.M.Glasg., appointed Assistant Medical Officer at the Barony Parish Asylum at Woodilee Lenzie. D. C. Rees, M.R.C.S., L.R.C.P., appointed Senior House-Surgeon to the Seamen's (late "Dreadnought") Branch Hospital, Royal Albert Docks, E. William R. Smith, M.D., B.S.Lond., F.R.C.S.Eng., appointed House Surgeon to the Nottingham General Hospital. J. M. G. Swainson, L.R.C.P., M.R.C.S., appointed Resident Obstetric Assistant to the Westminster Hospital. W. E. Thomas, M.D., appointed Medical Officer for the Pentre and Ystrad Rhondda District of the Pontypridd Union. William Thomson, appointed Surgeon in Dublin to the London and North-Western Railway Company. G. S. Warburton, M.R.C.S., L.S.A., appointed Medical Officer and Public Vaccinator to the Upper Ystradyfodwg District of the Pontypridd Union. Brian Watts, M.D.Brux., M.R.C.S.Eng., L.R.C.P.Lond., appointed a Resident Medical Office Sheffield General Infirmary.

### VACANCIES.

London Homœopathic Hospital.—Resident Medical Officer. Appointment for twelve months, six months as House Surgeon and six months as House Physician. Salary £80 per annum, with board and apartments. Applications to the Secretary-Superintendent by September 30th.

### PASS LISTS.

### University of Edinburgh.

Second Professional Examination for Degrees in Medicine and Surgery.—The following is the official list of candidates who have completed this examination: A. L. Anderson, W. N. Barker (with distinction), E. F. Bashford, F. P. Bester, S. C. Bose, E. R. Branch, A. E. Brookes, R. Bruce, E. H. Brunt, H. M. Bundey, C. J. Caddick, I. D. Cameron, R. Cameron, R. Carswell, A. I. S. Coghill, E. F. Coghlan, R. Crawford, F. P. Crowther, E. F. Cyriax, G. Drummond, H. Duckworth, C. L. Dunn, R. W. T. Ewart, S. Faed, G. W. Fitzgerald, J. G. Forbes, A. Fordyce, C. Forsyth, A. F. Fraser, E. Frost, R. Gellatly, A. B. George, J. F. Gibbon, H. E. Gibbs, H. A. H. Gilmer, F. J. Gray, J. M. Gray, A. A. Gunn, A. G. Hamilton, G. Hamilton, J. Hunter, J. W. Ingles, C. King (with distinction), F. O. Lasbrey, T. C. Lauder, Evariste Laval, J. Leggate, P. A. Leighton, L. S. L. Liddell, A. W. Limont, A. M. Little, W. Lockerbie, J. S. Low, P. L. M'All, A. J. M'Clymont, M. R. M'Dougall, A. C. M'Gilchrist, M.A. (with distinction), A. M. M'Intosh, W. M'Kay, E. C. G. Maddock, M. B. Marshall, Ruth Massey, C. A. Mayne, W. P. Meldrum, C. W. F. Melville, L. H. B. Mills, J. Monroe, B. E. Myers, B. K. Nariman, T. M. Ness, M.A.; A. S. Parker, F. M. Parry, G. H. Parry, L. D. Parsons, H. K. Paxton, H. R. Phillips, T. Pretsell, F. W. Price, W. Raine, J. M. Reid, J. H. Rhodes (with distinction), F. W. Rigby, J. R. Roberts, B.Sc.; G. A. Rorie, J. E. Rosenstein, A. Shearer, A. B. Shed, J. A. Shoolbread, E. Somerville, J. F. Stewart, W. Tarr, W. E. Tellett, W. B. Thain, A. H. Thomas, A. H. Thompson, P. G. Leeb-du-Toit, A. K. Traill, G. R. Twomey, R. C. Verley, H. Wade, W. E. J. Wallis, D. B. Waters, M.A.; A. E. B. Wood, and A. H. Wood.

Third Professional Examination for the Degrees of M.B. and Ch.B.—The following is the official list of candidates who have passed this examination: A. J. T. Allen, H. L. S. D. Belasco, L. Boyer, W. S. Eaton, W. H. Fowler, G. Fourie, W. Gorrie, W. G. Heath, A. H. Keun, J. W. M'Intosh, W. E. M'Kechnie, G. W. Miller, E. F. M. Neave, J. H. P. Paton, H. K. Paxton, A. H. Pirie, C. M. Robertson, G. H. Stewart, and C. P. B. Wall.

